# Juvenile psammomatoid ossifying fibroma of the maxilla and mandible: A systematic review of published case reports

**DOI:** 10.1002/cre2.687

**Published:** 2022-11-03

**Authors:** Chandini R., Saranya R., Khadijah Mohideen, Murali Balasubramaniam, Snehashish Ghosh, Safal Dhungel

**Affiliations:** ^1^ Department of Oral Pathology Sathyabama Dental College and Hospital Chennai India; ^2^ Department of Oral Pathology College of Medical Sciences Bharatpur Nepal; ^3^ Department of Oral and Maxillofacial Surgery College of Medical Sciences Bharatpur Nepal

**Keywords:** fibroma, juvenile, ossifying, psammomatoid

## Abstract

**Objective:**

The aim of this study is to evaluate recent evidence‐based data that summarize the clinicopathological findings and treatment along with follow‐up measures taken in terms of published cases of Juvenile psammomatoid ossifying fibroma (JPOF) of the maxilla and mandible by a systematic review.

**Materials and Methods:**

The databases searched were PubMed, MEDLINE, Scopus, Google scholar, and Cross references. Only those case reports of JPOFs published in the English language from 2000 to 2022 were considered. All cases included confirmed JPOF lesions histopathologically. The SR‐included details like clinical and radiographic data, follow‐up details such as recurrence, and the presence of any adverse outcome.

**Results:**

The database search produced 595 articles from 2000 to 2022, among which 22 case reports were included in the systematic review. The mean age of JPOF occurrence in patients was 18 ± 16 years. A male predilection was noted among patients younger than 14 years of age, whereas a female predilection was noted in patients older than 14 years of age. Frequent involvement of the mandible (56%) compared to the maxilla (44%) was reported. The posterior mandible was the most commonly affected site involving numerous adjacent structures. The expansile nature of the JPOF displayed 57% buccolingual expansion, 50% downward displacement or erosion of the lower border of the mandible and 81% of  involvement of the maxillary antrum/pterygoid plate/orbital floor. Among the 20 cases reported, the treatment provided included surgical excision in 45% of the patients, jaw resection in 35% of the patients, and enucleation and curettage in 18% of the patients. Follow‐up details were provided in 80% of the reports that showed recurrence.

**Conclusions:**

The diagnosis of JPOF requires correlation of the clinical and radiographic features with key histopathological features. Although long‐term follow‐up of the case reports has been reported, the data lack information about the long‐term outcomes of JPOF.

## 1 INTRODUCTION

Juvenile psammomatoid ossifying fibroma (JPOF) is considered to be a benign fibro‐osseous lesion. Since the local aggressive behavior of JPOF parallels that of a malignant entity, it is also referred to as “aggressive psammomatoid ossifying fibroma.” Unlike most benign fibro‐osseous lesions, JPOF presents predominantly in a younger age group of patients aged 16–33 years. JPOF frequently occurs in the paranasal sinus and orbital region. Its involvement in the gnathic bones is relatively uncommon (Neville et al., 2015; Waknis et al., 2011). The natural history of JPOF is still debated due to its unique clinical, histopathological, and behavioral patterns. Immunohistochemical (IHC) analysis shows positivity for osteonectin (bone‐specific protein), which indicates an osseous origin (Sarode et al., 2011). An alternative origin proposed for cementum‐producing JPOF is the periodontal ligament; however, there is a lack of substantial evidence to confirm this. The histopathology of JPOF consists of characteristic psammomatoid bodies embedded in a highly cellular stroma. Psammomoid bodies are spherical calcifications resembling psammoma bodies. Early aggressive surgical intervention is of utmost importance as JPOF of the craniofacial complex frequently invades the cranial base and the orbit (Sarode et al., 2011). Hence, the objective of this systematic review was to evaluate recent available data for evidence that summarizes the clinicopathological findings, treatment, and follow‐up measures used in published cases of JPOF of the maxilla and mandible.

## 2 AIM

The principal aim of this study was to include the maximum number of studies and to evaluate the principal clinicopathological features of JPOF by SR and its aggressive nature and assess the recurrence rate of the lesion.

## 3 MATERIALS AND METHODS

The search approach followed the SR procedure for other oral and maxillofacial lesions. The search design, the strategy for citing the literature, and the interpretation of the data retrieved are described below. The present systematic review was conducted according to the PRISMA guidelines.

### 3.1 Search strategy

Databases like MEDLINE, PubMed, Scopus, and Cross‐reference were used for the literature search. The search terminologies used were a combination of (i) juvenile psammomatoid ossifying fibroma and mandible AND (ii) juvenile psammomatoid ossifying fibroma and maxilla, (iii) aggressive psammomatoid ossifying fibroma AND maxilla (iv) aggressive psammomatoid ossifying fibroma AND mandible. Boolean operators such as “OR” and “AND” were used in combination during the search.

**Figure 1:**
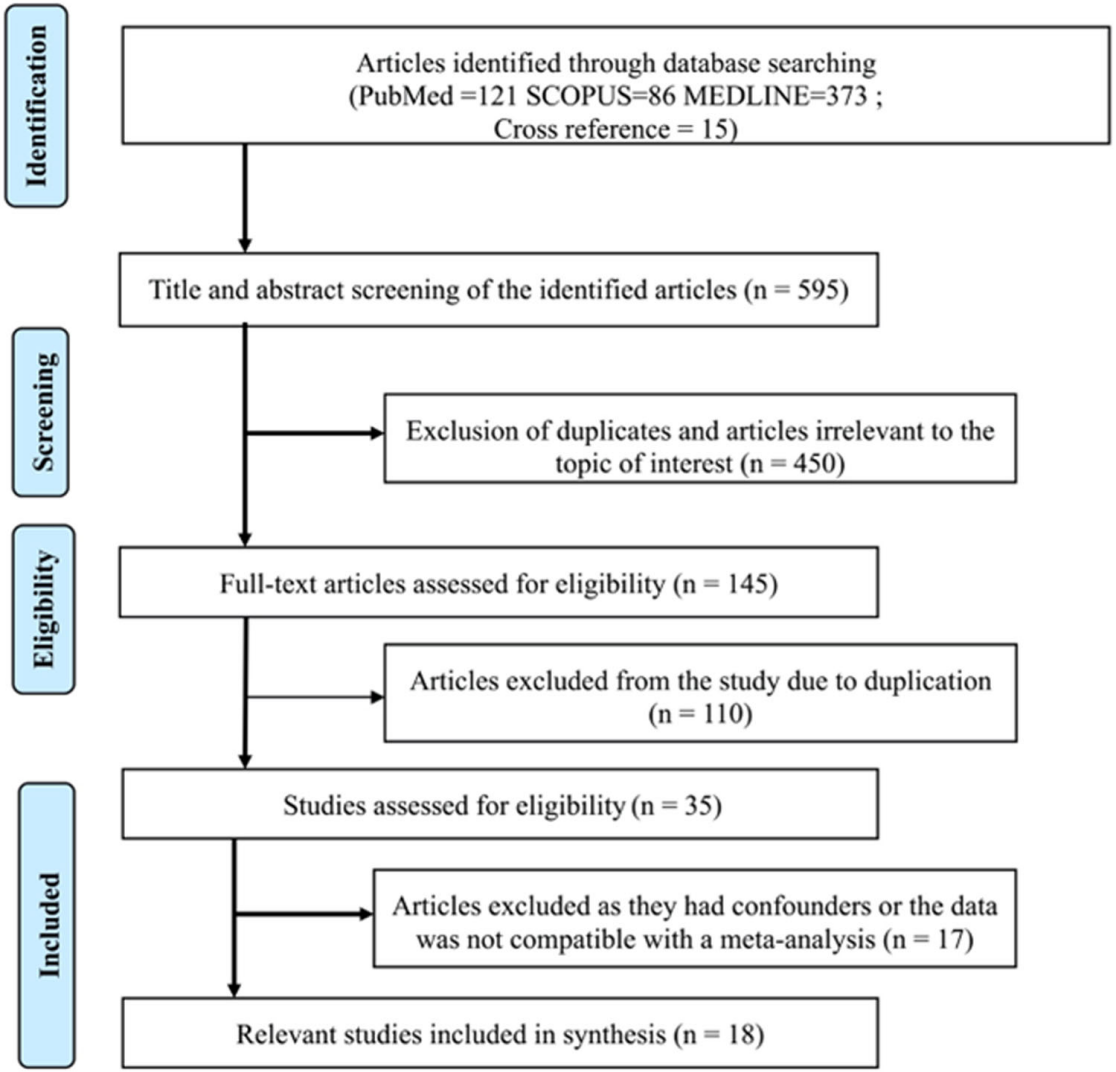
Flow chart of the study selection process

This review aimed to assess the clinicopathological findings and treatment measures used to treat JPOF of the maxilla and mandible. Finally, we included quantitative and qualitative research performed globally and published in English in a peer‐reviewed journal from 2000 onwards. The selection process of the article is shown in Figure 1.

There was inclusion (Criteria 1–5) and exclusion (Criteria 1‐6) criteria for the SR. Each report was evaluated under these criteria in strict sequence.

Inclusion criteria for inclusion were as follows: (i) case reports and case series, (ii) articles in the English language, (iii) case reports on juvenile psammomatoid ossifying fibroma, (iv) anatomical site—maxilla and mandible, and (v) complete description of cases. There was no restriction on sex, location, and ethnicity. Exclusion criteria were as follows: (i) inappropriate design, including review articles, original studies, editorials, letters to the editor, and conference abstracts, (ii) studies not in the English language, (iii) any other ossifying fibroma that is not of a psammomatoid variant, (iv) region other than the maxilla or mandible, (v) cases with insufficient clinical, radiological, and histopathological details, and (vi) exclusion of referred cases.

We additionally scanned the reference lists of the included research for any extra articles. The articles obtained from the databases were compiled using the EndNote reference manager. Two unbiased reviewers performed the title and summary screening, full‐textual content screening, and refined assessment. Disagreements were resolved by discussion and consensus.

The risk of bias was assessed using the JBI critical appraisal checklist for case reports and is shown in Table 1 (Joanna Briggs Institute, 2017, p. 3).

**Table 1:** Risk of bias assessment

	Study		Age radiographic and histopathological features				Demographics	Clinical history	Follow‐up	Clinical features			
S. No	Author (year)	Q1	Q2	Q3	Q4	Q5	Q6	Q7	Q8	Q9	Q10	% yes	Risk
1	Betina and Manas (2017)	NA	+	+	NA	NA	+	×	×	+	NA	40	High
2	Deshingkar et al. (2014)	NA	+	+	NA	NA	+	+	×	+	NA	50	Moderate
3	Foss and Fielding (2007)	NA	+	+	NA	NA	+	+	×	+	NA	50	Moderate
4	Gantala et al. (2015)	NA	+	+	NA	NA	+	+	+	+	NA	60	Moderate
5	Gotmare et al. (2017)	NA	+	+	NA	NA	+	+	+	+	NA	60	Moderate
6	Halama et al. (2013)	NA	+	+	NA	NA	+	+	+	+	NA	60	Moderate
7	LMG Figueiredo et al. (2014)	NA	+	+	NA	NA	+	+	+	+	NA	60	Moderate
8	Malathi et al. (2011)	NA	+	+	NA	NA	+	+	×	+	NA	50	Moderate
9	Manjunatha et al. (2016)	NA	+	+	NA	NA	+	+	+	+	NA	60	Moderate
10	Melo et al. (2014)	NA	+	+	NA	NA	+	+	+	+	NA	60	Moderate
11	Nair et al. (2010)	NA	+	+	NA	NA	+	+	+	+	NA	60	Moderate
12	Patigaroo (2011)	NA	+	+	NA	NA	+	+	+	+	NA	60	Moderate
13	Patil et al. (2013)	NA	+	+	NA	NA	+	+	+	+	NA	60	Moderate
14	Cárdenas‐Perilla et al. (2016)	NA	+	+	NA	NA	+	×	×	+	NA	40	High
15	Rahmani et al. (2020)	NA	+	+	NA	NA	+	+	+	+	NA	60	Moderate
16	Dandriyal et al. (2012)	NA	+	+	NA	NA	+	+	×	+	NA	50	Moderate
17	Sarode et al. (2018)	NA	+	+	NA	NA	+	+	+	+	NA	60	Moderate
18	Tamgadge et al. (2014)	NA	+	+	NA	NA	+	+	+	+	NA	60	Moderate
19	Tolentino et al. (2012)	NA	+	+	NA	NA	+	×	×	+	NA	40	High
20	Turin et al. (2019)	NA	+	+	NA	NA	+	+	+	+	NA	60	Moderate
21	Yadav et al. (2013)	NA	+	+	NA	NA	+	+	+	+	NA	60	Moderate
22	Yang et al. (2009)	NA	+	+	NA	NA	+	+	+	+	NA	60	Moderate

*Note*: Q1–Q10 indicate questions 1–10 based on the JBI Critical Appraisal Checklist for Case reports. The risk of bias was ranked as High when the study received up to 49% of “Yes” scores, moderate when the study received from 50%–69% of “yes” scores, and low when the study received more than 70% of “yes” scores. “+” indicates yes, “×” indicates no and “NA” indicates not applicable.

Abbreviation: JBI, Joanna Briggs Institute.

## 4 RESULTS

The database search produced 590 articles spanning from 2000 to 2022. Databases like PubMed, Medline, Scopus, and Google scholar revealed 121, 373, 86, and 10 articles, respectively, along with 15 additional reports identified by cross reference harvesting. The titles and abstracts of 605 reports were reviewed, among which 450 were rejected for the following reasons: reviews, retrospective analysis, abstracts rather than case reports, and lesions reported to be present on other sites. The remaining 145 reports were then subjected to the selection criteria in strict sequence. The repetitive reports and articles that lacked complete patient details or did not provide a definitive diagnosis of the psammomatoid variant of ossifying fibroma were automatically rejected.

### 4.1 Analysis of SR

Finally, 22 reports were included in the present SR. The distribution of the clinical, decade of presentation, and radiological, histological, and surgical details with follow‐up were extracted from each report and are summarized in Table 2.

**Table 2:** Summary of study articles

S. No	Author (year)	Age in years/gender	Location	Radiology	Histopathology	Treatment	Follow‐up
1.	Betina and Manas (2017)	41/M	Left anterior mandible	Radiolucent lesion	Highly cellular lesional tissue comprising of plump hyperchromatic fibroblasts and numerous basophilic calcifications consisting of central basophilic areas with peripheral eosinophilic areas resembling psammomatoid bodies	NA	NA
2.	Deshingkar et al. (2014)	18/M	Right para‐symphyseal region to the posterior border of the ramus of the mandible and superioinferior from the preauricular region to the inferior border of the body of the mandible	Multilocular expansile lesion on the left side of the body and ramus of the mandible without invasion of the coronoid and condylar processes. Presence of wispy radiopacities, with endosteal scalloping and a narrow transitional zone with the adjacent normal bone.	Bland fibroblastic spindle cells interspersed with numerous irregular and spherical ossicles showing varying degrees of calcification with minimal extracellular collagen deposition. These psammoma body‐like ossicles were relatively acellular, with peripheral eosinophillic rimming and distributed homogeneously.	Hemi‐mandibulectomy with disarticulation of the condyle, followed by a titanium reconstruction plate with a condylar head	NA
3.	Foss and Fielding (2007)	4/M	Right posterior mandible	Well‐demarcated unilocular lesion of the right posterior mandible. There was circumferential expansion of the mandible with sparing of the condyle. The body of the lesion showed a relatively homogeneous ground glass, but subtly variegated, radiodense appearance. The characteristic narrow radiolucent rim between the residual osseous cortex and the body of the lesion.	Highly cellular stroma showing numerous fibroblastic cells interspersed with numerous irregular and spherical ossicles showing varying degrees of calcification. These psammoma body‐like ossicles were relatively acellular, with a concentric pattern of lamination.	Surgical resection of the mass via hemi‐mandibulectomy	NA
4.	Gantala et al. (2015)	15/M	Right maxilla	Primarily radiolucent with irregular and scattered calcifications	Highly cellular stroma consisting of numerous plump fibroblasts arranged in streaming fascicles. Few psammoma‐like ossicles with peripheral brush borders were also noted.	Enucleation done	15 months postsurgery, the surgical site showed optimal healing
5.	Gotmare et al. (2017)	8/M	painless swelling on the left side of the mandible	Large multilocular radiolucent lesion on the left side of the mandible with thinning of the inferior border of the mandible	Fibrocellular stroma with oval to spindle‐shaped cell proliferation surrounding the areas of ossifications and calcifications. Calcifications are basophilic in the center surrounding the eosinophilic border resembling psammoma bodies. It also showed multiple sinusoidal blood‐filled spaces devoid of endothelial lining and surrounded by fibrocellular stroma.	Hemi‐mandibulectomy along with the fixation of a construction plate	no sign of recurrence after 6‐month of follow‐up
6.	Halama et al. (2013)	7/F	Left maxilla, pterygoid process	Mixed radiopaque–radiolucent mass	Histopathological features were compatible with JPOF	Surgical excision with reconstruction	Postoperative long‐term follow‐up was advised
7.	Figueiredo et al. (2014)	4/M	Recurrent lesion in the left mandible after 8‐month of follow‐up of fibrous dysplasia	A multilocular radiolucent area, infiltrative, cortical destructive aspect, in the left side of the mandible	Highly cellular stroma consisting of spindle cells and irregular psammomatoid cementicles with osteoid rims	Osteotomy and cryotherapy	No sign of recurrence after 18‐month of follow‐up
8.	Malathi et al. (2011)	46/F	Right maxillary posterior tooth region	Unilocular radiolucent lesion with areas of radiopacity	Cellular fibroblastic stroma containing spherical and curved ossicles. Concentric lamellated ossicles resembling psammoma bodies were present. The periphery of these ossicles showed a brush border that was blending into the surrounding stroma.	Partial maxillectomy	NA
		31/F	Molar region of the left mandible	Well‐demarcated unilocular radiolucent lesion interspersed with areas of radiopacity	Cellular fibroblastic stroma containing spherical and curved ossicles. Concentric lamellated ossicles resembling psammoma bodies were present. The periphery of these ossicles showed a brush border that was blending into the surrounding stroma.	NA	NA
9.	Manjunatha et al. (2016)	6/M	Right body of the mandible	Large radiolucency with an intact inferior border of the mandible on the right side	Highly cellular lesional tissue comprising of plump hyperchromatic fibroblasts and numerous spherical structures were basophilic in the center and eosinophilic in the periphery, termed psammoma bodies	Surgical enucleation	3‐month of follow‐up with no evidence of recurrence
10.	Melo et al. (2014)	35/F	Left mandibular ramus	Expansile osteolytic lesion	Histopathological features were compatible with JPOF	Mandibulectomy, followed by reconstruction	5‐month of follow‐up and no recurrence
11.	Nair et al. (2010)	15/F	Swelling in the left malar area	Maxillary lateral topographic occlusal view, which showed expansion of the buccal cortical plate and diffuse radiopacity in the molar region. The PNS view showed circumscribed radiopacity occupying almost the whole of the maxillary sinus with well‐defined borders.	Highly fibrocellular mesenchymal tissue containing abundant spherical and globular calcified masses or ossicles. The mesenchymal cells are mostly plump and spindle shaped	Surgical excision	No recurrence at the 6‐month follow‐up
12.	Patigaroo (2011)	20/F	Right maxilla, nasal cavity	Expansile osteolytic lesion	Highly cellular stroma consisting of spindle‐shaped cells arranged in the form of strands and whorls. Osteoblast‐rimmed osteoid strands and small psammoma‐like bodies were also noted.	Total maxillectomy	Obturator was placed 3 weeks postsurgery
13.	Patil et al. (2013)	7/M	Right posterior region of the mandible	Large radiolucency and well‐defi ned sclerotic border along with radiopacity observed in the center of the lesion (mixed lesion) with the inferior border intact	Histopathological features were compatible with JPOF	Excision and curettage without resection	3‐year of follow‐up and no recurrence
		15/F	Right maxilla, maxillary sinus	Expansile osteolytic lesion			
14.	Cárdenas‐Perilla et al. (2016)	7/F	Left maxilla, maxillary sinus	Expansile osteolytic lesion	Immature bony trabeculae and psammomatoid body embedded in a highly cellular background	NA	NA
15.	Rahmani et al. (2020)	11/M	Left maxilla, maxillary sinus, nasal, and infraorbital regions	A ground glass lesion measuring 3 × 3 × 2.8 cm involving the left frontal bone with extension to the orbital roof	Tumor well demarcated from the surrounding native bone, composed of fibroblastic stroma containing small ossicles (psammomatoid bodies). The ossicles had a thick irregular osteoid rim and coalesced at the periphery to form irregular thin bony trabeculae	Surgical excision with reconstruction	18 months postsurgery, no recurrence was detected
16.	Dandriyal et al. (2012)	14/F	Right mandible	Well‐defined radiopacities surrounded by radiolucency with a sclerotic border	Histopathological features were compatible with JPOF	Surgical excision with reconstruction	NA
17.	Sarode et al. (2018)	10/M	Anterior maxilla	Ground glass appearance with ill‐defined borders over the anterior region of the maxilla extending to the left quadrant. CT showed an expansile lesion involving the alveolar arch of the left maxilla and extending into the premaxillary region and the hard palate.	Highly cellular lesion with spherical ossicles were acellular and had a basophilic center and an eosinophilic peripheral fringe resembling psammoma bodies	Maxillectomy followed by reconstruction	Follow‐up for past 3 years with no recurrence
18.	Tamgadge et al. (2014)	7/M	Left ramus of the mandible	Expansile lesion in the left ramus of the mandible extending up to the cortical plates	Proliferation of oval to spindle‐shaped cells along with spherical mineralized ossicles resembling psammoma bodies that were basophilic at the center and eosinophilic at the periphery with brush border with multiple sinusoidal spaces	Enucleation and curettage	2‐month follow‐up and no recurrence
19.	Tolentino et al. (2012)	20/M	Right body of the mandible	Multilocular osteolytic lesion with significant expansion of both cortical plates with well‐defined limits, located on the right mandibular body	Cell‐rich connective tissue containing numerous acellular spherical ossicles with basophilic concentric laminations and an osteoid rim, characterizing psammoma bodies	NA	NA
		12/M	Right mandible	Expansive, multilocular osteolytic lesion with expansion of both cortical plates, extending from the distal aspect of the second premolar to the anterior border of the ramus.	Cell‐rich fibrous tissue containing mineralized material consisting of psammoma body‐like spherical ossicles. These bodies showed concentric lamella with basophilic centers that were acellular or had sparse cells.	Surgical excision and vigorous curettage, followed by reconstruction	15‐month of follow‐up with no recurrence
20.	Turin et al. (2019)	11/F	Left maxilla	Large expansile osseous lesion involving the left maxilla with a predominant hyperdense, ground‐glass‐appearing component, with associated dense bone and cystic change	Cellular fibroblastic stroma containing uniformly distributed ossicles resembling psammoma bodies	Surgical excision	4‐month of follow‐up and no recurrence
21.	Yadav et al. (2013)	14/F	Right mandible	Mixed radiolucent–radiopaque lesion with well‐defined borders and thinning of the lower border of the mandible	Highly cellular lesion with spherical ossicles were acellular and had a basophilic center and an eosinophilic peripheral fringe resembling psammoma bodies	Surgical excision with reconstruction	6‐month of follow‐up with no recurrence
22.	Yang et al. (2009)	46/F	Right maxilla, maxillary sinus, nasal, and infraorbital regions	Well‐defined lesion with diffuse calcification	Highly cellular stroma consisting of spindle cells and irregular psammomatoid cementicles with osteoid rims	Surgical excision	15 months postsurgery, no recurrence was detected

Abbreviations: NA, indicates not applicable; PNS, paranasal sinus.

The range of cases covered was significantly from the year 2000 to 2022. During and before 2015,  16 cases were reported and 9 cases were reported after 2015. Among the 22 case reports, the patients' age range was 4–46 years, with an average age of 18 ± 16 years. 56% of the patients were below or equal to 14 years of age, and the rest, 44%, were older than 14 years of age.

The gender distribution of the patients is shown in Table 2, with 12 females and 13 males in total. Before 2015, eight female patients were reported and after 2015, four female patients were reported, whereas eight male patients were reported before 2015 and five male patients were reported after 2015. On comparing age with gender, 36% of females and 64% males younger than 14 years of age were reported. Similarly, 63% of females and 37% males older than 14 years of age were reported. Based on these data, a male predilection among patients younger than 14 years of age and a female predilection among patients older than 14 years of age were observed.

Based on the location of the lesion particular to the jaw the specific case reports were included. Of the cases with the jaw involved, 56% of the lesions were present in the mandible and 44% were present in the maxilla. 45% of cases were reported in the right quadrant and 55% in the left half of the maxilla. In the mandible, 57% of cases were reported in the right quadrant and the rest, 43% in the left. Both the maxilla and the mandible showed a posterior jaw predilection.

The radiographic presentation of the case reports was analyzed based on OPG and CT scan findings. A multilocular shape was found in 14% of cases in the reports of Deshingkar et al. (2014). In the remaining cases (86%), unilocular lesions were reported. The expansile nature of the JPOF was indicated by buccolingual expansion (57%), downward displacement or erosion of the lower border of the mandible (50%), involvement of the maxillary antrum/pterygoid plate/orbital floor (81%) with tooth displacement (32%), and root resorption (19%). The treatment provided in 20 reported cases included surgical excision in 45% of the patients, jaw resection in 35% of the patients, and enucleation and curettage in 18% of the patients.

Although 14 case reports were reported with some follow‐up details and the rest 8 were assumed to have also been followed up, none of these case reports displayed recurrence.

## 5 DISCUSSION

The fibro‐osseous lesion is characterized by the replacement of the normal bone architecture with fibro‐osseous tissue consisting predominantly of spindle‐shaped fibroblast‐like cells that are arranged in whorls or strands, with varying amounts of psammomatoid bodies and osseous matrix rimmed with osteoblasts (Patigaroo, 2011; Su et al., 1997). The biological nature of entities categorized under fibro‐osseous lesions ranges from self‐limiting hamartomas to aggressive benign neoplasms. Among the various fibro‐osseous lesions, ossifying fibroma affects the jaws and shows behavior varying from slow growth to occasionally aggressive local destruction (Wu et al., 1986). Most OFs grow slowly and do not recur after complete excision, but a minority, specifically in children, shows rapid growth and a tendency to recur; these are generally called “juvenile ossifying fibroma” (JOF) (Slootweg et al., 1994).

JPOF is one of the most aggressive types of fibro‐osseous lesions involving the craniofacial bones. Unlike conventional ossifying fibroma, the juvenile forms of ossifying fibroma have unique clinicopathological features. Some leeway was allowed to include all possible reports from different parts of the world to ensure the inclusion of maximum relevant reports. However, it becomes necessary to limit the number of such cases to 10% to minimize skewing of the SR's results just by analyzing the psammomatoid variant of juvenile ossifying fibroma and not considering the trabecular variant.

JPOF is present in a relatively younger age group ranging from 8 to 12 years in the case of the juvenile trabecular ossifying fibroma (JTOF) and 16–32 years in the case of JPOF (Neville et al., 2015). JPOF is relatively more common in the sinonasal tract and the orbit and rarely involves the gnathic bones. Both the juvenile forms of ossifying fibroma show aggressive growth patterns characterized by gross destruction of the involved bone, resulting in an expansile osteolytic lesion. Depending on the degree of bone destruction and the presence of mineralization, the radiographic pattern of JPOF ranges from a predominantly osteolytic (radiolucent) lesion to a mixed radiolucent–radiopaque lesion. JPOF frequently involves adjacent structures, especially the paranasal sinuses and the nasal cavity (Sarode et al., 2011; Waknis et al., 2011).

No prevalence data are available for JPOF, as it is a rare entity. However, it needs to be kept in mind by the clinicians as a differential diagnosis (El‐Mofty, 2002). Juvenile psammomatoid ossifying fibroma does not have the same natural course as Fibrous Dysplasia (FD) (Noffke, 1998), and there is no suitable radiological criterion to distinguish between psammomatoid and trabecular variants of juvenile ossifying fibroma. JPOF may acquire an incorrect diagnosis until the tissue is available for pathologic examination. The histopathological presentation of numerous basophilic calcifications consisting of central basophilic areas with peripheral eosinophilic areas resembling psammomatoid bodies was used for the diagnosis of JPOF.

Like conventional ossifying fibroma, the histopathology of JPOF consists of varying degrees and forms of mineralization. JPOF shows a relatively higher degree of cellularity than its conventional counterpart, as evidenced in our present case. The characteristic differentiating feature between JTOF and JPOF is the presence of psammomatoid bodies in the latter. Although several cases of malignant transformation of fibro‐osseous lesions have been reported, it is not reported in JPOF.

An aggressive clinical course, occurrence in a relatively younger age group, an expansile radiolucent/mixed radiographic presentation, and a varying degree of mineralization embedded in a highly cellular stroma are some key clinicopathological features used to differentiate the juvenile form of ossifying fibroma from other fibro‐osseous lesions. In addition to the features mentioned above, psammomatoid bodies or a predominant trabecular pattern aid in making the final diagnosis as JPOF or JTOF, respectively (Sarode et al., 2011).

The overall management strategy for JPOF is drastically different than that for FD. There is debate regarding management with curettage or enucleation. The specific well‐circumscribed lesions may be amenable to treatment with these techniques (J. Han et al., 2016). These techniques may allow salvage of the involved bony or neurovascular structures that an aggressive resection would otherwise sacrifice (Abuzinada & Alyamani, 2010; Slootweg et al.,^1994^). It should be noted that the rates of recurrence do appear to be higher when more conservative resections are performed (J. Han et al., 2016). JPOF frequently encroaches onto adjacent bony structures, complicating surgical intervention (Sarode et al., 2011). Radical resection is considered the standard treatment for JPOF (Foss & Fielding, 2007; M. H. Han et al., 1991), with recurrence being common, ranging from 30% to 56% (Makek, 1983; Margo et al., 1985). Because of the aggressive clinical course and the high recurrence rate of JPOF, early intervention with radical surgery is required (Sarode et al., 2011). Recurrence is hypothesized to be caused by the infiltrative nature of the tumor leading to incomplete excision (El‐Mofty, 2002). Though the literature describes high recurrence rates for more conservative treatment, no recurrences were reported in the present systematic review regardless of the type of resection chosen.

It is noteworthy that a careful examination and correlations of clinical, radiological, and histopathological features are essential to arrive at the correct diagnosis, which plays a vital role in the management of patients.

## 6 CONCLUSION

JPOF has unique clinicopathological features. The diagnosis of JPOS requires correlating the clinical and radiographic features with key histopathological features, such as psammomatoid bodies and varying degrees of mineralization in a highly cellular stroma. Given the different management algorithms, correct tissue diagnosis is crucial to provide appropriate treatment. They show aggressive local invasion into adjacent structures, causing difficulty in removing the lesion “in toto,” resulting in a high recurrence rate. It is essential to closely follow‐up JPOF cases for a long time due to the associated high recurrent rate for aggressive lesions.

## AUTHOR CONTRIBUTIONS

Chandini R. and Snehashish Ghosh contributed to the design of the study, search and selection, and drafted the manuscript. Saranya R. and Murali Balasubramaniam contributed to the study and Snehashish Ghosh contributed to the analysis and interpretation and critically revised the manuscript. Khadijah Mohideen, Safal Dhungel, and Snehashish Ghosh contributed to the conceptualization and design of the study, search and selection, analysis and interpretation, and critically revised the manuscript. All the authors gave final approval and agreed to be accountable for all aspects of the work, ensuring integrity and accuracy.

## CONFLICT OF INTEREST

The authors declare no conflict of interest.

## Data Availability

Data analyzed in this study included a reanalysis of existing data, which are cited in the reference section.

## References

[cre2687-bib-0001] Abuzinada, S. , & Alyamani, A. (2010). Management of juvenile ossifying fibroma in the maxilla and mandible. Journal of Maxillofacial and Oral Surgery, 9(1), 91–95. 23139579 10.1007/s12663-010-0027-6 PMC3453706

[cre2687-bib-0002] Betina, C. , & Manas, B. (2017). Psammomatoid juvenile ossifying fibroma of mandible in a 41‐year male patient. Journal of the College of Physicians and Surgeons Pakistan, 27(1), 49–50. 28292370

[cre2687-bib-0003] Cárdenas‐Perilla, R. , Santamaria, C. , & Muñoz‐Acosta, J. M. (2016). Juvenile psammomatoid ossifying fibroma: Findings on bone scan. Clinical Nuclear Medicine, 41(9), 718–721. 27405032 10.1097/RLU.0000000000001297

[cre2687-bib-0004] Dandriyal, R. , Pandit, N. , Rao, S. , Sapra, G. , Sharma, H. , & Agarwal, U. (2012). Psammomatoid juvenile aggressive ossifying fibroma of mandible. National Journal of Maxillofacial Surgery, 3(1), 47. 23251058 10.4103/0975-5950.102155 PMC3513809

[cre2687-bib-0005] Deshingkar, S. A. , Barpande, S. R. , & Bhavthankar, J. D. (2014). Juvenile psammomatoid ossifying fibroma with secondary aneurysmal bone cyst of mandible. The Saudi Journal for Dental Research, 5(2), 135–138.

[cre2687-bib-0006] El‐Mofty, S. (2002). Psammomatoid and trabecular juvenile ossifying fibroma of the craniofacial skeleton: Two distinct clinicopathologic entities. Oral Surgery, Oral Medicine, Oral Pathology, Oral Radiology, and Endodontology, 93(3), 296–304. 10.1067/moe.2002.121545 11925539

[cre2687-bib-0007] Figueiredo, L. M. G. , de Oliveira, T. F. L. , Paraguassú, G. M. , de Hollanda Valente, R. O. , da Costa, W. R. M. , & Sarmento, V. A. (2014). Psammomatoid juvenile ossifying fibroma: Case study and a review. Oral and Maxillofacial Surgery, 18(1), 87–93. 23435579 10.1007/s10006-013-0400-y

[cre2687-bib-0008] Foss, R. D. , & Fielding, C. G. (2007). Juvenile psammomatoid ossifying fibroma. Head and Neck Pathology, 1(1), 33–34. 20614278 10.1007/s12105-007-0001-x PMC2807504

[cre2687-bib-0009] Gantala, R. , Vemula, A. Y. , Kubbi, J. R. , Sekhar, M. M. , & Jhawar, D. (2015). Psammomatoid juvenile ossifying fibroma involving upper jaw: A rare case report. Journal of Clinical and Diagnostic Research: JCDR, 9(7), ZD17. 10.7860/JCDR/2015/14603.6199 PMC4573055 26393222

[cre2687-bib-0010] Gotmare, S. S. , Tamgadge, A. , Tamgadge, S. , & Kesarkar, K. S. (2017). Recurrent psammomatoid juvenile ossifying fibroma with aneurysmal bone cyst: An unusual case presentation. Iranian Journal of Medical Sciences, 42(6), 603–606. 29184270 PMC5684383

[cre2687-bib-0011] Halama, D. , Hierl, T. , Wickenhauser, C. , & Pausch, N. (2013). Psammomatoid juvenile ossifying fibroma of the maxilla: Radical surgery with maxillary resection in a 7‐year‐old girl. Klinische Pädiatrie, 225(07), 418–419. 24166092 10.1055/s-0033-1355392

[cre2687-bib-0012] Han, J. , Hu, L. , Zhang, C. , Yang, X. , Tian, Z. , Wang, Y. , Zhu, L. , Yang, C. , Sun, J. , Zhang, C. , Li, J. , & Xu, L. (2016). Juvenile ossifying fibroma of the jaw: A retrospective study of 15 cases. International Journal of Oral and Maxillofacial Surgery, 45(3), 368–376. 26740351 10.1016/j.ijom.2015.12.004

[cre2687-bib-0013] Han, M. H. , Chang, K. H. , Lee, C. H. , Seo, J. W. , Han, M. C. , & Kim, C. W. (1991). Sinonasal psammomatoid ossifying fibromas: CT and MR manifestations. AJNR. American Journal of Neuroradiology, 12(1), 25–30. 1899514 PMC8367571

[cre2687-bib-0014] Joanna Briggs Institute . (2017). *Critical appraisal checklist for systematic reviews and research syntheses*. https://jbi.global/sites/default/files/2019-05/JBI_Critical_Appraisal-Checklist_for_Systematic_Reviews2017_0.pdf

[cre2687-bib-0015] Makek, M. (1983). Clinical pathology of fibro‐osteo‐cemental lesions in the cranio‐facial and jaw bones. Karger Publishers.

[cre2687-bib-0016] Malathi, N. , Radhika, T. , Thamizhchelvan, H. , Ravindran, C. , Ramkumar, S. , Giri, G. , & Gopal, D. (2011). Psammomatoid juvenile ossifying fibroma of the jaws. Journal of Oral and Maxillofacial Pathology, 15(3), 326. 22144839 10.4103/0973-029X.86710 PMC3227263

[cre2687-bib-0017] Manjunatha, B. , Purohit, S. , Kiran, S. , & Mahita, V. (2016). Psammomatoid juvenile ossifying fibroma of mandible in a 6‐year‐old child. Indian Journal of Dentistry, 7(1), 44. 27134454 10.4103/0975-962X.179370 PMC4836097

[cre2687-bib-0018] Margo, C. E. , Ragsdale, B. D. , Perman, K. I. , Zimmerman, L. E. , & Sweet, D. E. (1985). Psammomatoid (juvenile) ossifying fibroma of the orbit. Ophthalmology, 92(1), 150–159. 3974992 10.1016/s0161-6420(85)34070-8

[cre2687-bib-0019] Melo, A. R. , Vasconcelos, B. C. , Carneiro, S. C. , do Amaral, M. F. , da Fonte Neto, A. S. , & Cardoso, M. S. (2014). Juvenile psammomatoid ossifying fibroma with massive mandibular involvement. General Dentistry, 62(5), 34–36. 25184712

[cre2687-bib-0020] Nair, P. , Kumar, A. , Hegde, K. , & Neelakantan, S. (2010). Psammomatoid juvenile ossifying fibroma: A case report with literature review. Journal of Indian Academy of Oral Medicine and Radiology, 22(5), S53–S57.

[cre2687-bib-0021] Neville, B. W. , Damm, D. D. , Allen, C. M. , & Chi, A. C. (2015). Oral and maxillofacial pathology. Elsevier Health Sciences.

[cre2687-bib-0022] Noffke, C. E. (1998). Juvenile ossifying fibroma of the mandible. Dento Maxillo Facial Radiology, 27(6), 363–366. 10895636 10.1038/sj/dmfr/4600384

[cre2687-bib-0023] Patigaroo, S. A. (2011). Juvenile psammomatoid ossifying fibroma (JPOF) of maxilla—A rare entity. Journal of Maxillofacial and Oral Surgery, 10(2), 155–158. 22654369 10.1007/s12663-010-0065-0 PMC3177524

[cre2687-bib-0024] Patil, R. , Chakravarthy, C. , Sunder, S. , & Shekar, R. (2013). Psammomatoid type juvenile ossifying fibroma of mandible. Annals of Maxillofacial Surgery, 3(1), 100–103. 23662272 10.4103/2231-0746.110081 PMC3645601

[cre2687-bib-0025] Rahmani, M. , Hendi, K. , Dalfardi, S. , Larijani, A. , & Alimohamadi, M. (2020). Juvenile psammomatoid ossifying fibroma of the orbital roof: A rare cause of proptosis among children. Pediatric Neurosurgery, 55(3), 163–168. 32756056 10.1159/000508691

[cre2687-bib-0026] Sarode, S. C. , Sarode, G. S. , Ingale, Y. , Ingale, M. , Majumdar, B. , Patil, N. , & Patil, S. (2018). Recurrent juvenile psammomatoid ossifying fibroma with secondary aneurysmal bone cyst of the maxilla: A case report and review of literature. Clinics and Practice, 8(3), 1085. 30090219 10.4081/cp.2018.1085 PMC6060481

[cre2687-bib-0027] Sarode, S. C. , Sarode, G. S. , Waknis, P. , Patil, A. , & Jashika, M. (2011). Juvenile psammomatoid ossifying fibroma: A review. Oral Oncology, 47(12), 1110–1116. 21840246 10.1016/j.oraloncology.2011.06.513

[cre2687-bib-0028] Slootweg, P. J. , Panders, A. K. , Koopmans, R. , & Nikkels, P. G. (1994). Juvenile ossifying fibroma. An analysis of 33 cases with emphasis on histopathological aspects. Journal of Oral Pathology & Medicine: Official Publication of the International Association of Oral Pathologists and the American Academy of Oral Pathology, 23(9), 385–388. 7823298 10.1111/j.1600-0714.1994.tb00081.x

[cre2687-bib-0029] Su, L. , Weathers, D. R. , & Waldron, C. A. (1997). Distinguishing features of focal cemento‐osseous dysplasia and cemento‐ossifying fibromas. Oral Surgery, Oral Medicine, Oral Pathology, Oral Radiology, and Endodontology, 84(5), 540–549. 10.1016/s1079-2104(97)90271-7 9394387

[cre2687-bib-0030] Tamgadge, S. , Avinash, T. , Bhalerao, S. , & Rajhans, S. (2014). Juvenile psammomatoid ossifying fibroma with aneurysmal bone cyst in the posterior mandible. Ecancermedicalscience, 8, 471. 25374619 10.3332/ecancer.2014.471 PMC4203472

[cre2687-bib-0031] Tolentino, E. S. , Centurion, B. S. , Tjioe, K. C. , Casaroto, A. R. , Tobouti, P. L. , Frederigue Junior, U. , Lara, V. S. , Damante, J. H. , Sant'ana, E. , & Gonçales, E. S. (2012). Psammomatoid juvenile ossifying fibroma: An analysis of 2 cases affecting the mandible with review of the literature. Oral Surgery, Oral Medicine, Oral Pathology and Oral Radiology, 113(6), e40–e45. 10.1016/j.oooo.2011.08.005 22668716

[cre2687-bib-0032] Turin, S. Y. , Purnell, C. , & Gosain, A. K. (2019). Fibrous dysplasia and juvenile psammomatoid ossifying fibroma: A case of mistaken identity. The Cleft Palate‐Craniofacial Journal, 56(8), 1083–1088. 30813749 10.1177/1055665619833294

[cre2687-bib-0033] Waknis, P. , Sarode, S. C. , & Dolas, R. S. (2011). Psammomatoid juvenile ossifying fibroma of the mandible with secondary aneurysmal bone cyst: A case report. Asian Journal of Oral and Maxillofacial Surgery, 23(2), 83–86.

[cre2687-bib-0034] Wu, P. C. , Leung, P. K. Y. , & Ma, K. (1986). Recurrent cementifying fibroma. Journal of Oral and Maxillofacial Surgery, 44(3), 229–234. 3456447 10.1016/0278-2391(86)90114-x

[cre2687-bib-0035] Yadav, N. , Gupta, P. , Naik, S. , & Aggarwal, A. (2013). Juvenile psammomatoid ossifying fibroma: An unusual case report. Contemporary Clinical Dentistry, 4(4), 566. 24403813 10.4103/0976-237X.123094 PMC3883348

[cre2687-bib-0036] Yang, H. Y. , Zheng, L. W. , MDS, J. L. , Yin, W. H. , Yang, H. J. , & Zwahlen, R. A. (2009). Psammomatoid juvenile cemento‐ossifying fibroma of the maxilla. Journal of Craniofacial Surgery, 20(4), 1190–1192. 19553841 10.1097/SCS.0b013e3181acdcb2

